# A nickel nanoparticle engineered CoFe_2_O_4_@GO–Kryptofix 22 composite: a green and retrievable catalytic system for the synthesis of 1,4-benzodiazepines in water†

**DOI:** 10.1039/d0ra01671c

**Published:** 2020-04-16

**Authors:** Roya Mozafari, Mohammad Ghadermazi

**Affiliations:** Department of Chemistry, University of Kurdistan P. O. Box 66135-416 Sanandaj Iran mghadermazi@yahoo.com +98 87 33624133 +98 87 33624133

## Abstract

A composite of Ni nanoparticles incorporated in Kryptofix 22 conjugated magnetic nano-graphene oxide, CoFe_2_O_4_@GO–K 22·Ni, was synthesized *via* the grafting of Kryptofix 22 moieties on the magnetic nano-graphene oxide surface, followed by reaction of the nanocomposite with nickel nitrate. The Kryptofix 22 host material unit cavities can stabilize the Ni nanoparticles effectively and prevent their aggregation and separation from the surface. Characterization of the catalysts by FT-IR, FE-SEM, TGA, ICP, EDX, XRD, VSM and BET aided understanding the catalyst structure and morphology. This catalyst was efficiently applied for the synthesis of 1,4-benzodiazepine derivatives. The main advantages of the method are mild reaction conditions, inexpensive catalyst, it is environmentally benign, has high to excellent yields and shorter reaction times. This organometallic catalyst can be easily separated from a reaction mixture and was successfully examined for six runs with a slight loss of catalytic activity.

## Abstract 2

In this study, a competent and efficient methodology for the synthesis of benzodiazepine over magnetically retrievable novel CoFe_2_O_4_@GO–K 22 anchored Ni is reported.

## Graphical abstract

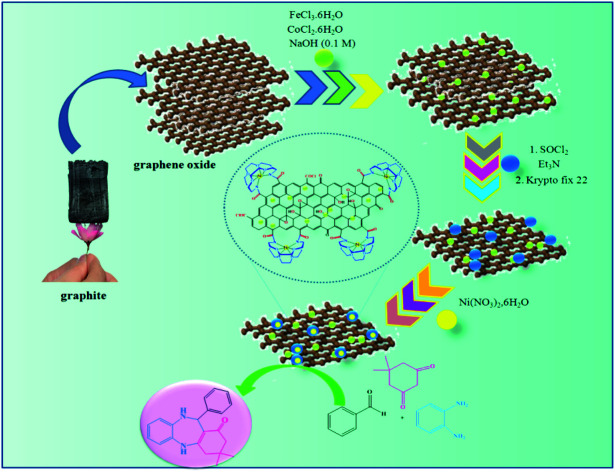

## 1. Introduction

Green chemistry is the study of the design and application of chemical products and processes to reduce or to eliminate negative impacts to human health and the environment, and where possible to utilize renewable raw materials. The development of green methods with high catalytic activity systems has received a great deal of research attention in organic synthesis for environmental and economic reasons.^1–5^ The aim of the field of catalysis in terms of green chemistry is to develop environmentally benign, practical, clean, economical and efficient processes for catalyst separation and recycling. To achieve these goals, graphene oxide (GO) has been widely explored to replace conventional catalysts or use as supporting material for improving the performance of catalysts due to its large specific surface area, unique layered structure, high thermal stability, intrinsic mechanical, electrical properties, and excellent flexibility.^6–9^ However, the expensive and tedious separation and recovery of powdered GO is a barrier for its widespread industry-scale applications. To control these problems, magnetic separation techniques is a fast, simple, economical, and green approach, which makes removing and reusing of the catalyst is possible without the need to lengthy, cumbersome and expensive centrifuge.^10–13^ Therefore, the magnetic GO nanocatalysts are regarded as ideal supports for the heterogenize the homogeneous catalysts support due to their unique properties such as simple procurement, good dispersion properties, excellent catalytic activity, outstanding stability, selectivity and well separation of the catalyst *via* an external magnet.^14,15^ Among metal ferrite nanoparticles, cobalt ferrite (CoFe_2_O_4_) have gained a great deal of attention due to their moderate saturation magnetization, high chemical stability and mechanical hardness. CoFe_2_O_4_ nanoparticles decorated on graphene oxide to obtain CoFe_2_O_4_@GO composite, will achieve further stabilization of these nanoparticles, and also prevents formation of aggregates in solution.^16–19^

**Fig. 1:**
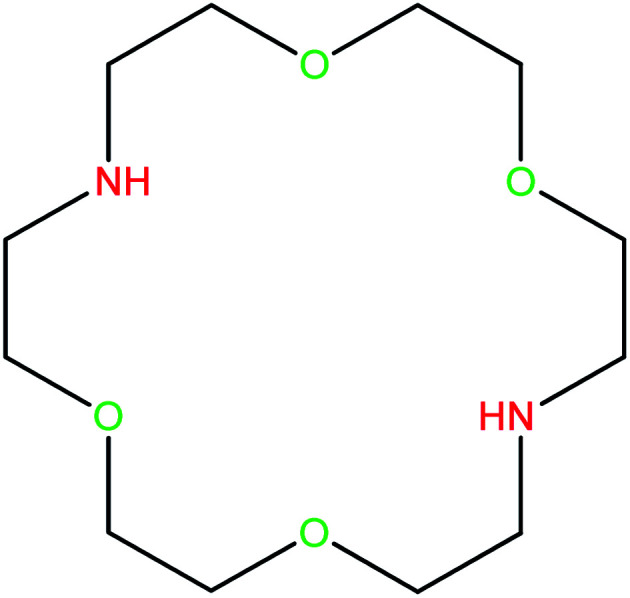
The structure of Kryptofix 22 (K 22).

On the other hand, Kryptofix 22 (K 22) as a specific class of aza-crown ether have been known for their high affinity and selectivity to bind transition metals (Fig. 1). The unique structure and properties of K 22 have made them a popular choice for a wide range of applications over the past few decades. They have been widely utilized in many disciplines such as supramolecular chemistry, biochemistry, materials science, catalysis, separation and biomedicine. They are exceptionally versatile in selectively binding a range of metal ions, providing development of the area of host–guest chemistry.^20–25^

The incorporation of metal onto support material has received significant attention due to the sustainable green chemistry uses it presents. Nevertheless, we anchored nickel nanoparticles incorporated Kryptofix 22 onto the surface of magnetic nano-graphene oxide as efficient and recyclable nanocatalyst for the synthesis of 1,4-benzodiazepine.

Benzodiazepine derivatives represent one of the most active classes of heterocyclic possessing a wide spectrum of physiological and pharmacological properties.^26–28^

Recently, many methods have been reported for the preparation of benzodiazepines through two-component or three-component condensation including the use of [H-NMP][HSO_4_],^29^ La(OH)_3_,^30^ Gr@TiO_2_ NCs,^31^ NiO–SiO_2_ NCs.^32^ Despite the merits of these procedures, each of them suffers at least from one of the following limitations: low yields, unavailability of the reagents, long reaction times, high toxicity, operational costs, use of strong acids, harsh reaction conditions, and tedious workup procedures. The drawbacks mentioned above can be solved *via* the development of a more versatile and also environmentally friendly method. The present study was conducted to develop new, synthetic, and useful methodologies using organometallic based catalyst for the preparation of various biologically active heterocyclic compounds.

The aim of this presented work is to highlight the synergistic effects of the combined properties of highly mesoporous surface of magnetic nano-graphene oxide, and Kryptofix 22 cavity, in the trapping and stabilizing of the Ni nanoparticles and explored its application in the synthesis of 1,4-disubstituted benzodiazepines as a proficient, harmless to the environment, recyclable and magnetic powerful solid catalyst with good stability.

## 2. Experimental

### 2.1. Materials and physical measurements

All the chemicals and solvents used in this work were purchased from Merck and Sigma-Aldrich and were used without further purification. The morphology of nanocomposites was revealed by a scanning electron microscope (FESEM-TESCAN MIRA3). FT-IR spectra were taken on a PerkinElmer Spectrum Version10.4.4 spectrophotometer in KBr pellets and reported in cm^−1^. ^1^H NMR spectra were measured on a Bruker 400 MHz spectrometer in DMSO with chemical shift (*δ*) given in ppm. The TGA curve of the catalyst was recorded on a BAHR, SPA 503 at heating rates of 10 °C min^−1^. The X-ray powder diffraction (XRD) data were collected with Co Kα radiation (*λ* = *n*1.78897 Å) operating at *n*40 keV. VSM measurement was recorded by a Vibrating Sample Magnetometer (VSM) MDKFD. The size of the as-synthesized nanoparticle was determined by transmission electron microscopy (TEM) techniques using Zeiss-EM10C transmission electron. Energy-dispersive X-ray spectroscopy (EDX) analysis was obtained by MIRA3TESCANXMU instrument. Nitrogen adsorption measurements were conducted at 77.4 K on a Belsorp18. The specific surface area and the pore size distribution were calculated by Brunauer–Emmett–Teller method (BET) and Barrett–Joyner–Halenda (BJH) model, respectively. ICP analyzer (PerkinElmer, Optima 8300) was used for measuring the Ni loading of the catalyst.

### 2.2. Synthesis of graphene oxide (GO)

The graphene oxide was got ready according to the previously reported methods.^33,34^ The graphite powder (2.0 g) was treated with NaNO_3_ (1.0 g) in the cooled concentrated sulfuric acid (50 mL) under stirring in ice bath. Then, KMnO_4_ (7 g) was added slowly into the dispersion, and the mixture was stirred at lower than 15 °C. After 10 min, the ice bath was removed and the mixture was stirred at 40 °C for 6 h. In continue, deionized water (100 mL) was added under vigorous stirring and the diluted suspension was stirred at 90 °C for 30 min. Next step, hydrogen peroxide (30%, 7 mL) was added dropwise along with stirring to the mixture until the color of the reaction media was changed from black to the yellow. The solution was filtered and washed by HCl (5%) and deionized water several times to remove the excess of manganese and residual acid. The resulting GO solid was dried in air, at room temperature.

### 2.3. Synthesis of graphene oxide decorated with CoFe_2_O_4_ nanoparticles (CoFe_2_O_4_@GO)

Briefly, 1.0 g of dry GO was placed into a 150 mL round bottom flask, and 100 mL deionized water was added to the GO, and the mixture was sonicated for 45 min. To a magnetically stirred mixture of as-prepared dispersed GO, a solution of FeCl_3_·6H_2_O (0.8 mmol, 0.216 g), CoCl_2_·6H_2_O (0.4 mmol, 0.0516 g) in deionized water (15 mL) were added to the suspension of GO. The obtained solution was magnetically stirred at 80 °C for 45 min. After that, aqueous NaOH solution (0.1 M) was dropwise added to the reaction until the pH of the mixture solution was raised to 11. Eventually, the gained product was separated by a external magnet from the reaction media and washed with ethanol (50 mL × 3)/distilled water (50 mL × 3) mixture solution and then dried under vacuum at 60 °C for 12 h.

### 2.4. Synthesis of Kryptofix 22 conjugated magnetic nano-graphene oxide (CoFe_2_O_4_@GO–K 22)

In this step, CoFe_2_O_4_@GO (0.2 g) was added into the solution containing thionyl chloride (20 mL) and treated at 75 °C for 24 h. The gained product (CoFe_2_O_4_@GO-COCl) was collected by an external magnet and was washed with anhydrous dry tetrahydrofuran (THF) at least five times then dried at room temperature. During this process, CoFe_2_O_4_@GO-COCl (0.15 g) was first homogenized with dry DMF (20 mL) in an ultrasonic bath for 45 min and then a few drops of triethylamine (Et_3_N), and Kryptofix 22 (1.0 mmol, 0.26 g) were added to the round and the reaction mixture was sonicated for another 30 min and the stirring for another 12 h at 90 °C. The obtained product was washed with deionized water/ethanol successively and dried at 60 °C under vacuum. Finally, the Kryptofix 22 grafted graphene oxide product was obtained as a black powder.

### 2.5. Synthesis of Ni nanoparticles incorporated mesoporous magnetite graphene oxide (CoFe_2_O_4_@GO–K 22·Ni)

In the final step, CoFe_2_O_4_@GO–K 22·Ni was successfully prepared through coordination of CoFe_2_O_4_@GO–K 22 ligand to nickel. It should be noted that CoFe_2_O_4_@GO–K 22·Ni nanocatalyst was obtained by refluxing of Ni(NO_3_)_2_·6H_2_O (2.5 mmol, 0.730 g) with the CoFe_2_O_4_@GO–K 22 (1 g) in ethanol (25 mL) for 6 h. Ultimately, the resultant product was isolated by an external magnet and washed with ethanol/deionized water several times then dried at 60 °C.

### 2.6. General procedure for the synthesis of dibenzo-1,4-diazepine derivatives in the presence of CoFe_2_O_4_@GO–K 22·Ni system

In a round-bottom flask (25 mL) equipped with a magnetic stirrer and heater, dissolved aldehyde (1 mmol), dimedone (1 mmol, 0.140 g) and *o*-phenylenediamine (1 mmol, 0.108 g) CoFe_2_O_4_@GO–K 22·Ni (3 mg) in H_2_O (5 mL) was added. The blend was mixed at 60 °C for a suitable period of time (Table 1). Progress of the reaction was monitored by TLC (*n*-hexane/EtOAc, 10 : 3). After completion of the reaction, diethyl ether (10 mL) was added and the catalyst was removed by using an external magnet and washed with ethanol, vacuum dried, then subjected to the next run directly. The remaining solid product was recrystallized from aqueous ethanol to provide pure benzodiazepines. The final products data were specified by ^1^H and ^13^C NMR spectroscopy (see ESI†).

**Table 1:** Optimization of experimental conditions for the synthesis of dibenzo-1,4-diazepine derivatives under different conditions^a^

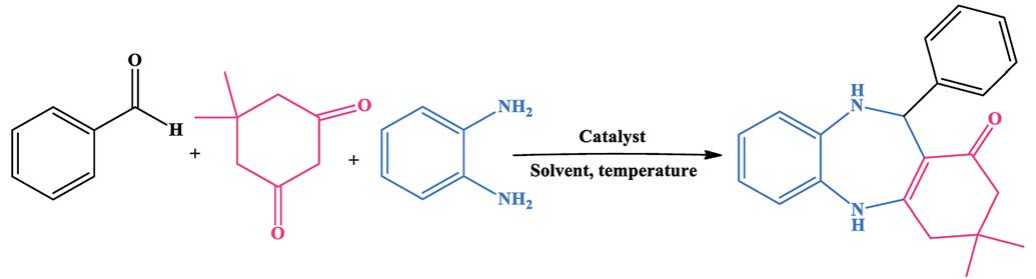					
Entry	Catalyst (mg)	Solvent (mL)	Temperature (°C)	Time (min)	Conversion
1	5	H_2_O	RT	60	60
2	4	H_2_O	RT	120	54
3	35	H_2_O	60	10	95
4	3	H_2_O	40	20	91
5	3	H_2_O	60	10	95
6	3	H_2_O	70	10	95
7	2	H_2_O	60	15	92
8	—	H_2_O	60	120	30
9	3	EtOH	60	30	88
10	3	CH_2_Cl_2_	60	60	45
11	3	EtOAc	60	60	68
12	CoFe_2_O_4_@GO	H_2_O	60	120	65

a Reaction conditions: 1 mmol of dimedone, *o*-phenylenediamine and benzaldehyde in 5 mL solvent.

## 3. Results and discussions

**Scheme 1:**
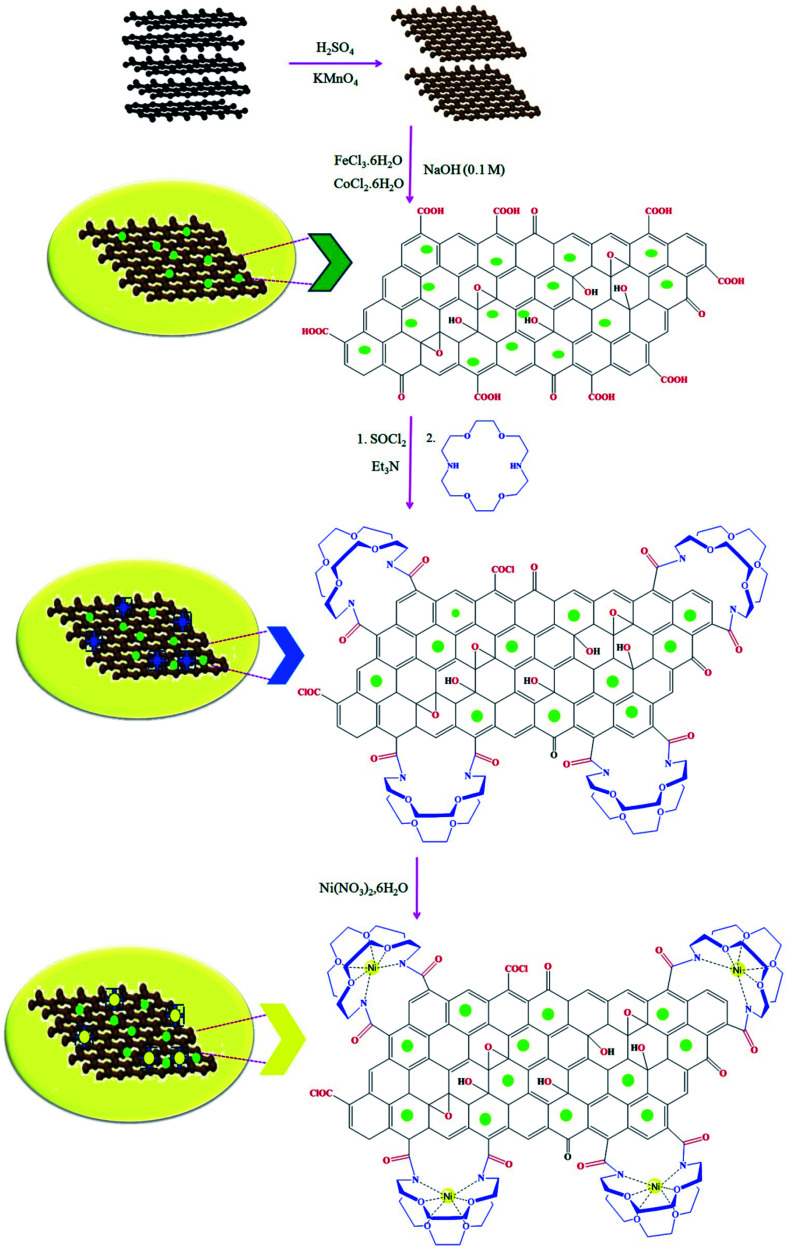
Step-by-step synthesis of CoFe_2_O_4_@GO–K 22·Ni.

The synthetic route for the preparation of the Ni nanoparticles incorporated Kryptofix 22 conjugated magnetic graphene oxide, CoFe_2_O_4_@GO–K 22·Ni, is outlined in (Scheme 1). The nanocomposite was prepared with a multistep process. Firstly, we started from conversion of graphite to GO and, then CoFe_2_O_4_ nanoparticles were deposited over the surface of graphene oxide by coprecipitation of Co^2+^ and Fe^3+^ ions in a basic aqueous solution followed by thermal treatment. The controllable nucleation site of CoFe_2_O_4_ on the GO can be realized by the addition of sodium hydroxide (NaOH) solution. The obtained CoFe_2_O_4_@GO was refluxed in SOCl_2_ to give the intermediate GO-COCl. Afterward, GO-COCl was reacted with Kryptofix 22 for the synthesis of Kryptofix 22-functionalized graphene oxide (CoFe_2_O_4_@GO–K 22). The scaffolds were synthesized by covalent linking of the carboxyl (–COCl) groups of GO with the amino (–NH) groups of Kryptofix 22. The treatment of CoFe_2_O_4_@GO–K 22 with Ni(NO_3_)_2_·6H_2_O provides CoFe_2_O_4_@GO–K 22·Ni through a stable interaction between the nickel and functional groups of the Kryptofix 22.

**Scheme 2:**
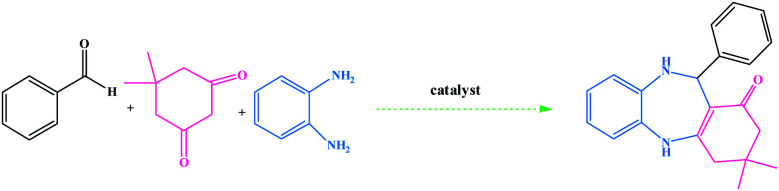
Synthesis of 1,4-benzodiazepine derivatives in the presence of CoFe_2_O_4_@GO–K 22·Ni nanocatalyst.

The method for the green and efficient synthesis of Ni nanoparticles stabilized by Kryptofix 22 conjugated magnetic nano-graphene oxide and investigatione of their catalytic activity for the synthesis 1,4-benzodiazepine derivatives in water are shown in (Scheme 2). This new nanocatalyst operates efficiently and safely in water and can be easily separated using an external magnet.

To characterize the nanocatalyst, and to confirm the immobilization of the active components on the pore surface of magnetic graphene oxide was characterized by various techniques such as FESEM, XRD, TGA, ICP-OES, EDX, BET, FT-IR and VSM.

### 3.1. Catalyst characterization

**Fig. 2:**
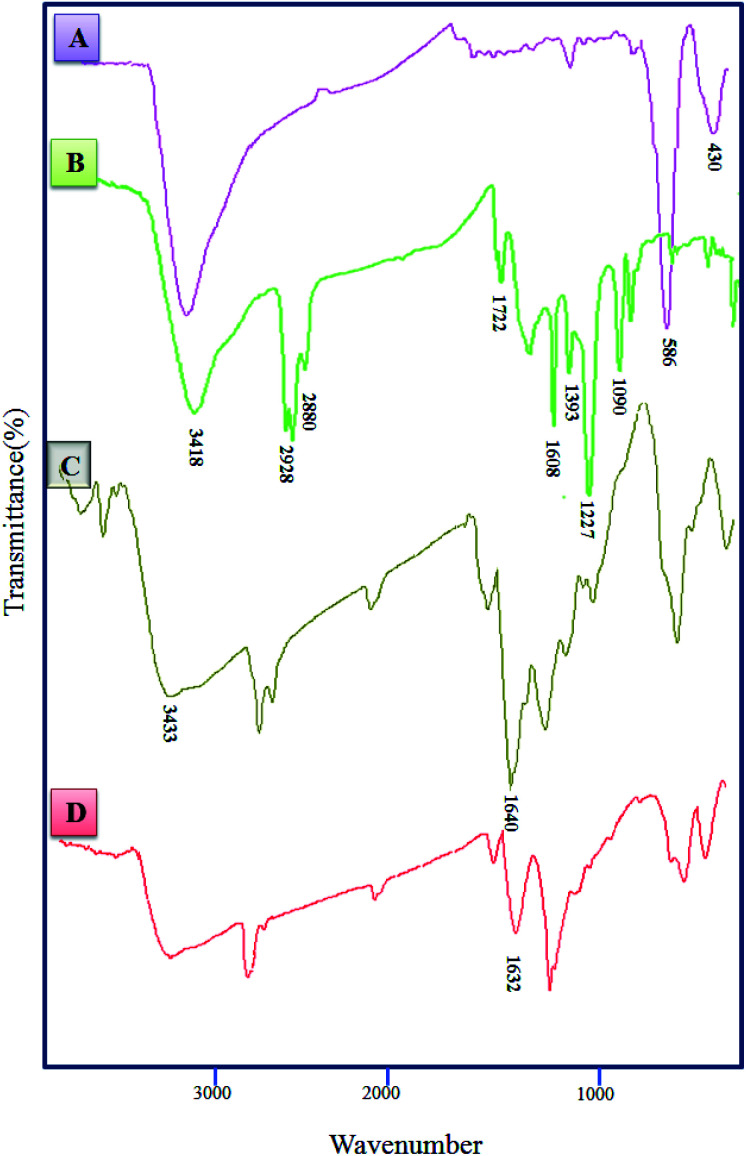
FT-IR spectra of CoFe_2_O_4_ (A), CoFe_2_O_4_@GO (B), CoFe_2_O_4_@GO–K 22 (C), CoFe_2_O_4_@GO–K 22·Ni (D).

Fig. 2 shows FT-IR spectra for CoFe_2_O_4_ (A), CoFe_2_O_4_@GO (B), CoFe_2_O_4_@GO–K 22 (C) and CoFe_2_O_4_@GO–K 22·Ni (D). In the spectrum of the CoFe_2_O_4_ two peaks at 430 cm^−1^ and 586 cm^−1^ are attributed to Fe–O stretching in the tetrahedral and octahedral sites of CoFe_2_O_4_, respectively.^35^ The band at 3418 cm^−1^ is assigned to the stretching vibration of the hydroxyl functional group (–OH) (Fig. 2A). The CoFe_2_O_4_@GO exhibited characteristic bands at 3418, 1722, 1608, 1393, 1227 and 1090 cm ^−1^ which have indicative of: O–H, carboxyl/carbonyl (C=O), aromatic (–C=C–), carboxyl (C–O), epoxy (–C–O), and alkoxy (–C–O) functional groups, respectively (Fig. 2B). In the FT-IR spectrum of CoFe_2_O_4_@GO–K 22, the peak at 1722 cm^−1^ was almost disappeared. Besides, a new broad band emerged at 1640 cm^−1^, corresponding to the C=O characteristic stretching band of the amide groups (Fig. 2C), that show K 22 was covalently grafted onto the surface of CoFe_2_O_4_@GO, also, the shift on spectrum to lower wave numbers belong to symmetrical and asymmetrical modes of the Kryptofix 22 bonds and metal is happened, that is due to a robust interaction between the O, N group of the nickel complex on the magnetic graphene oxide (Fig. 2D).

**Fig. 3:**
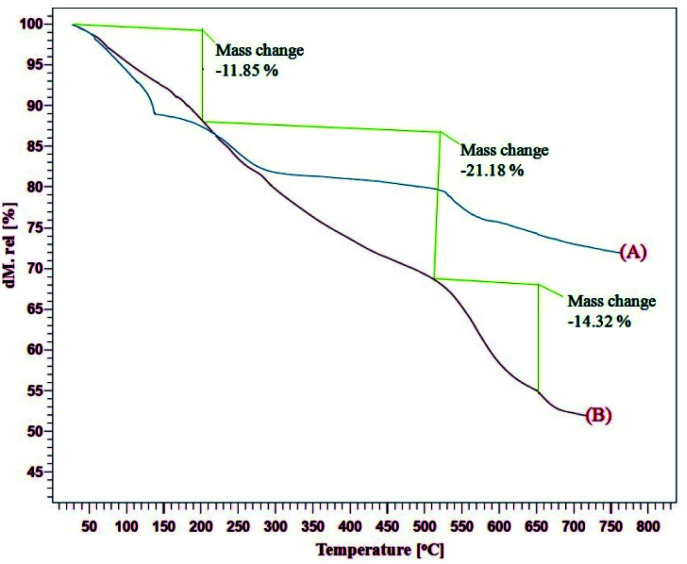
TGA curves of GO (A), CoFe_2_O_4_@GO–K 22·Ni (B) in air.

The TGA curves of the GO, CoFe_2_O_4_@GO–K 22·Ni indicates the weight loss of the organic material as they decompose upon heating (Fig. 3). Fig. 3 presents three weight loss steps in the TGA curve of the CoFe_2_O_4_@GO–K 22·Ni catalysts. The first weight loss (11.85%) between 20–220 °C due to the removal of adsorbed water moisture at the hybrid material surface of the mentioned catalyst is occurred. The next two weight losses (35.5%) from 210 to 660 °C are due to the decomposition and burning of GO and Kryptofix 22. On the basis of the results of the TGA curve the well grafting of GO and K 22 onto CoFe_2_O_4_ is verified.

**Fig. 4:**
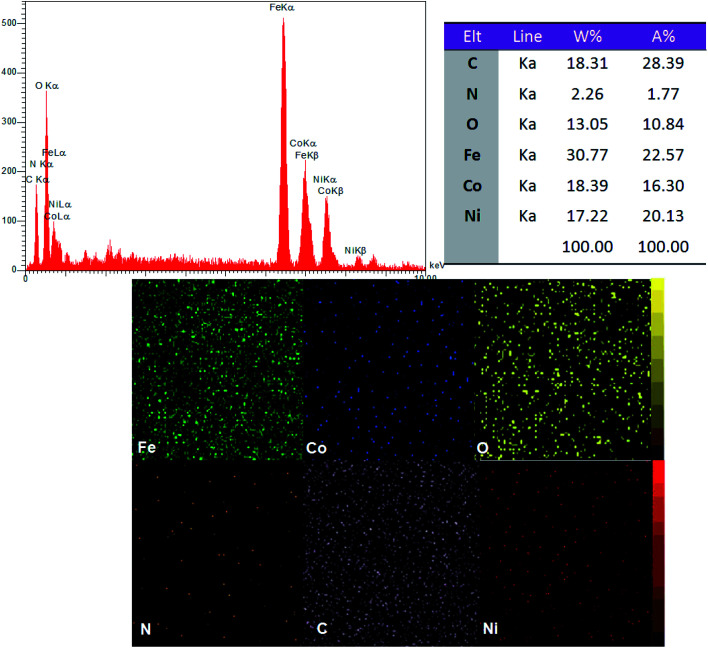
EDX spectrum and elemental mapping of CoFe_2_O_4_@GO–K 22·Ni nanocomposite.

EDX elemental analysis of the CoFe_2_O_4_@GO–K 22·Ni shows the existence of cobalt, iron, nitrogen, carbon, oxygen and nickel in the catalyst and the spectrum is depicted in Fig. 4. Also, the elemental mapping images indicate the uniform dispersion of Ni in the nanocomposite. This has been further confirmed from the EDX spectrum of the nanocatalyst.

Inductively ICP-OES analysis determined the exact amount of nickel loaded on graphene oxide–magnetite nanocomposite was found to be 0.41 mmol g^−1^.

**Fig. 5:**
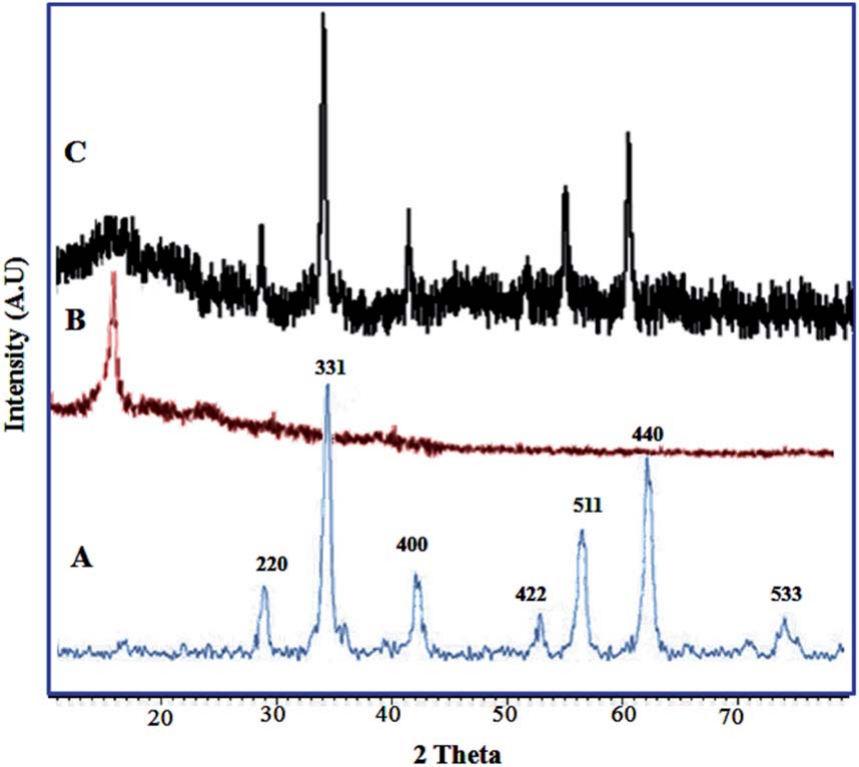
XRD patterns of CoFe_2_O_4_ (A), GO (B) and CoFe_2_O_4_@GO–K 22·Ni (C).

The XRD patterns of CoFe_2_O_4_, GO and CoFe_2_O_4_@GO–K 22·Ni are displayed in Fig. 5. The diffraction peaks related to Bragg's reflections from (2 2 0), (3 1 1), (4 0 0), (4 2 2), (5 1 1) and (4 4 0) planes correspond to the standard spinel structure of CoFe_2_O_4_ (JCPDS card no. 22-1086) (Fig. 5A). Based on the Debye–Scherrer equation, the average size of these CoFe_2_O_4_@GO–K 22·Ni particles is calculated to be nearly 23 nm. The parent graphene oxide exhibits a characteristic sharp peak at 2*θ* = 11.3° corresponding to the (001) reflection (Fig. 5B).^36^ Due to the fact that crystal growth of CoFe_2_O_4_ between the inter-layer of GO destroyed the regular layer stacking during sonochemical reaction, resulting in the exfoliation of GO and the disappearance of the (001) diffraction peak (Fig. 5C).

**Fig. 6:**
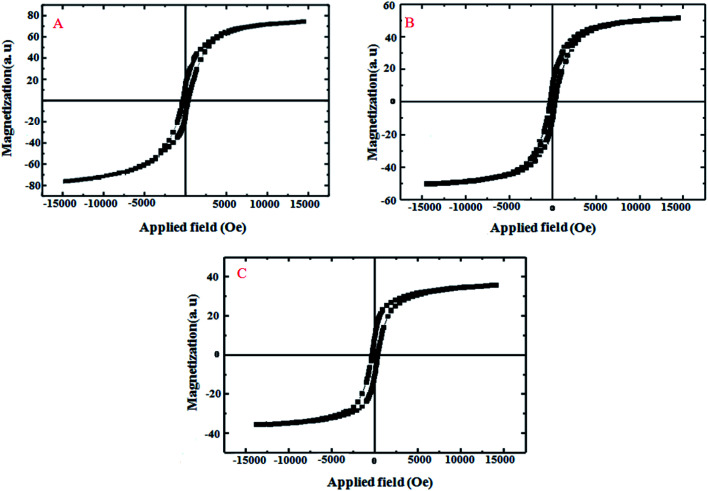
Magnetization curves of CoFe_2_O_4_ (A), CoFe_2_O_4_@GO (B) and CoFe_2_O_4_@GO–K 22·Ni (C).

As seen in Fig. 6, the characterization of the magnetic feature of CoFe_2_O_4_ (A), CoFe_2_O_4_@GO (B) and CoFe_2_O_4_@GO–K 22·Ni (C) were carried out using vibrating sample magnetometry (VSM) with a peak field of 15 kOe. It is clear from the hysteresis loops that the saturation magnetization (*M*_s_) of CoFe_2_O_4_, CoFe_2_O_4_@GO and CoFe_2_O_4_@GO–K 22·Ni are 79.05, 55.22, and 32.60 emu g^−1^, respectively. The decrease in mass saturation magnetization in the last two composites can be attributed to the decoration of COFe_2_O_4_ magnetic nanoparticles on GO and the grafting of K 22, Ni, respectively, over CoFe_2_O_4_. Although, a significant reduction was observed in the *M*_s_ values of the nanocatalyst, it is enough for any magnetic separation.

**Fig. 7:**
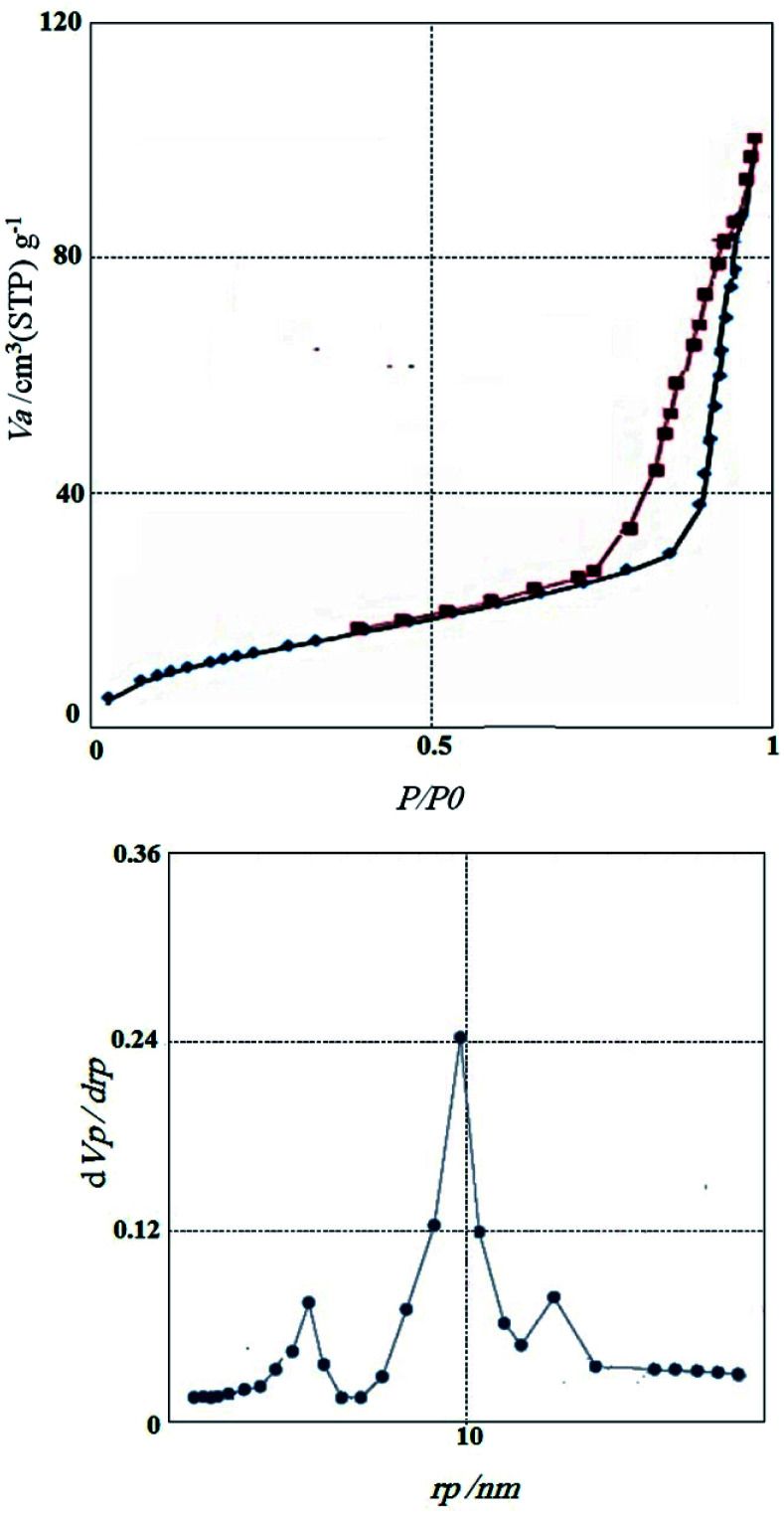
Nitrogen adsorption/desorption isotherms and BJH pore size distribution of CoFe_2_O_4_@GO–K 22·Ni.

Fig. 7 the nitrogen adsorption–desorption isotherms and pore size distributions of CoFe_2_O_4_@GO–K 22·Ni is illustrated. The materials had type IV isotherms, indicating that the mesostructure remained. According to Brunauer–Emmett–Teller (BET) analysis, the surface area, the pore volume, and the pore size of the catalyst is 109 m^2^ g^−1^, 0.143 cm^3^ g^−1^, 8.91 nm, respectively. Results indicate that immobilizing of K 22·Ni complex into the magnetic graphene oxide may be the reason of reduction the pore volume, pore size, and surface area of the catalysts.

**Fig. 8:**
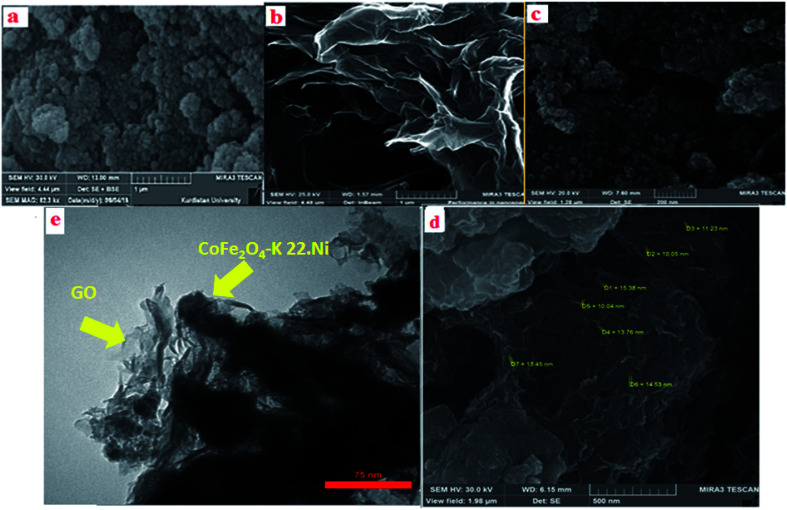
SEM images of CoFe_2_O_4_ (a), GO (b), CoFe_2_O_4_@GO (c), CoFe_2_O_4_@GO–K 22·Ni (d) and TEM image of CoFe_2_O_4_@GO–K 22·Ni (e).

The SEM is a useful method which is used to determine the morphology and size distribution of prepared nanoparticles. SEM images of the CoFe_2_O_4_, GO and CoFe_2_O_4_@GO–K 22·Ni catalyst are displayed in Fig. 8. As it is clearly seen, the CoFe_2_O_4_ spherical core–shell is structured with nano dimension ranging under 10 nm (Fig. 8a). Furthermore, as shown in the image CoFe_2_O_4_@GO–K 22·Ni comparing to the SEM image of GO, it was confirmed that some particles were anchored onto the surface of GO and the surface of GO became less transparent due to the presence of the CoFe_2_O_4_ nanoparticles and K22.Ni complex (Fig. 8d). Transmission electron microscopy (TEM) studies of the CoFe_2_O_4_@GO–K 22·Ni nanocomposite confirm that the nickel nanoparticles were incorporated in the CoFe_2_O_4_@GO–K 22 successfully (Fig. 8e).

We investigated catalytic activity of CoFe_2_O_4_@GO–K 22·Ni as a heterogeneous nanocatalyst in the synthesis of benzodiazepine in water as an easily available and green solvent as shown in Scheme 2.

To optimize the reaction conditions, we surveyed the synthesize of benzodiazepine on the basis of the reaction between dimedone, *o*-phenylenediamine and benzaldehyde as beginning materials under different reaction conditions. The results of these tests are summarized in Table 1.

Initially, the reaction was performed without any catalyst, but it did not proceed after a long time (Table 1, entry 8). The reaction proceeded with high speed and the corresponding products were isolated in excellent yields with 0.03 g of the catalyst (Table 1, entry 5).

To investigate the best reaction temperature, this procedure was studied at various temperatures and the best one was 60 °C (Table 1, entry 5). The reaction was repeated in different solvents such as water, ethanol, CH_2_Cl_2_, and EtOAc and it was found that water is more suitable for this reaction. Furthermore, the reaction was performed in the presence CoFe_2_O_4_@GO as catalyst, but it did not proceed after a long time. The obtained result confirmed that nickel nanoparticles play the main role of catalytic activity of the prepared CoFe_2_O_4_@GO–K 22·Ni for the synthesis of product.

To study the efficiency of this catalyst, benzaldehyde derivatives with electron releasing, and electron withdrawing groups were checked for the synthesis of 1,4-benzodiazepine derivatives. The results showed that both groups have high yields in short reaction time (Table 2).

**Table 2:** Synthesis of dibenzo-1,4-diazepine derivatives with CoFe_2_O_4_@GO–K 22·Ni system^a^

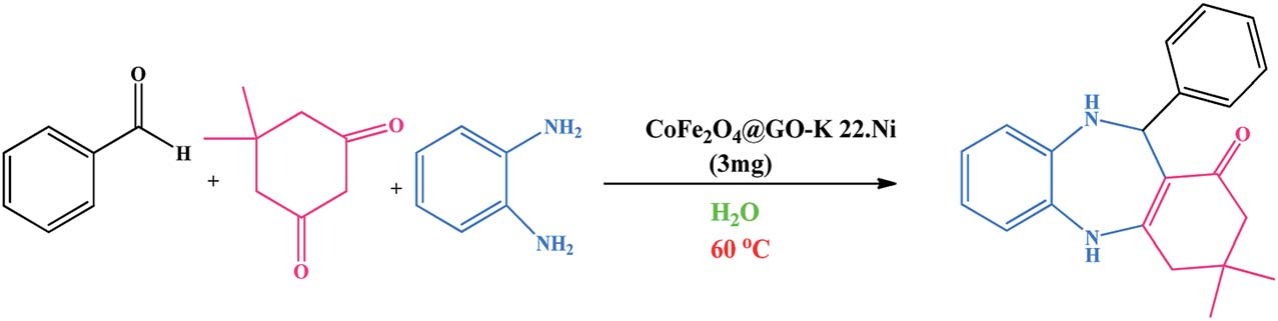					
Entry	Substrate	Product	Time (min)	Yield (%)	Mp/bp (°C) [lit.]
1	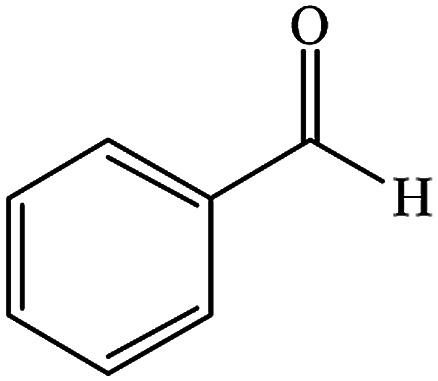	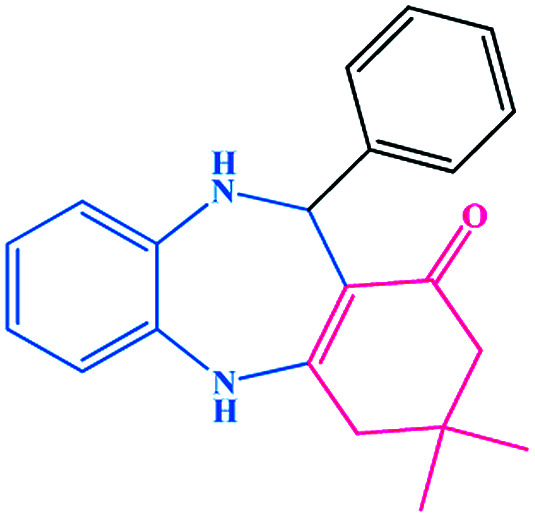	10	95	245–247 (ref. 29)
2	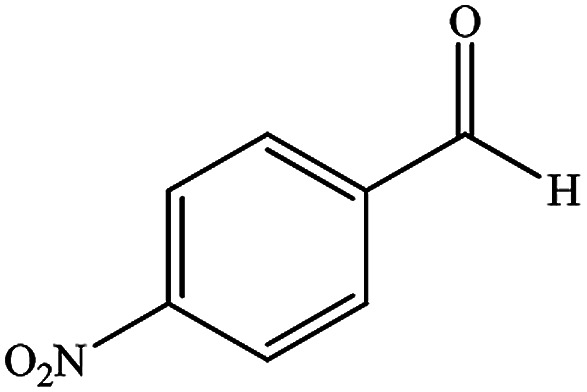	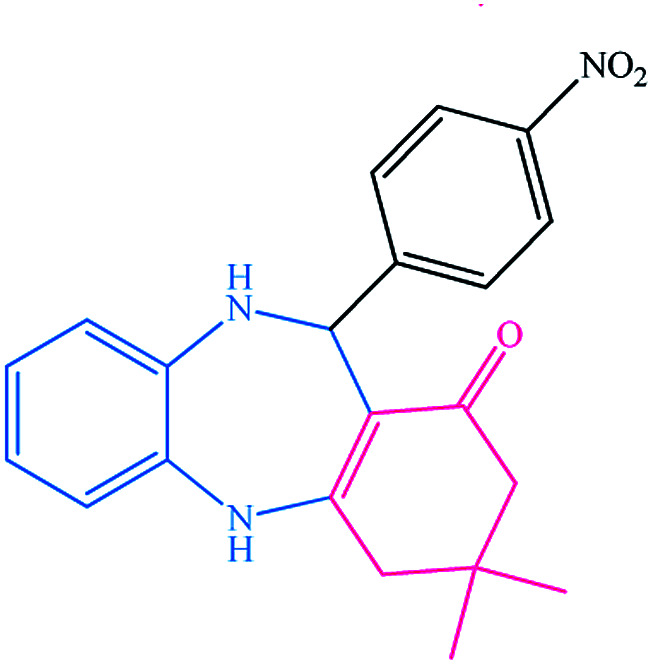	8	94	279–282 (ref. 37)
3	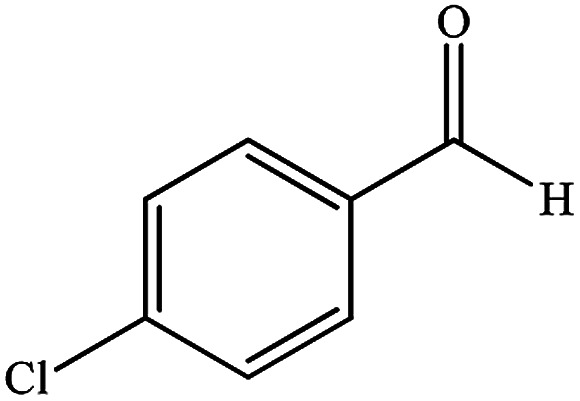	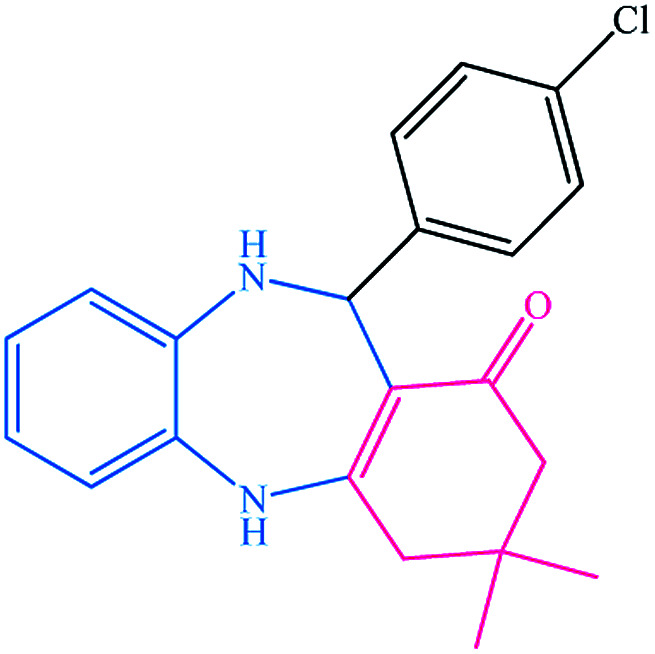	15	87	238–241 (ref. 38)
4	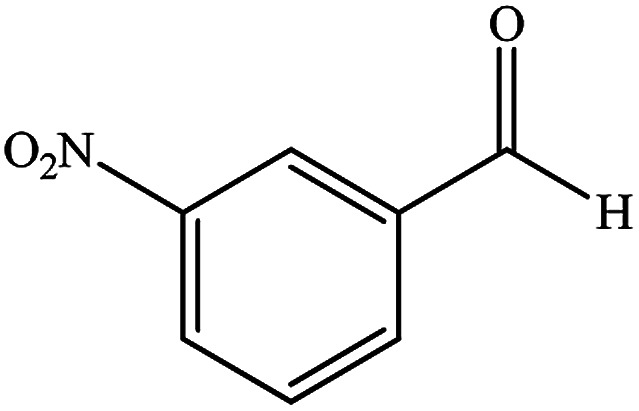	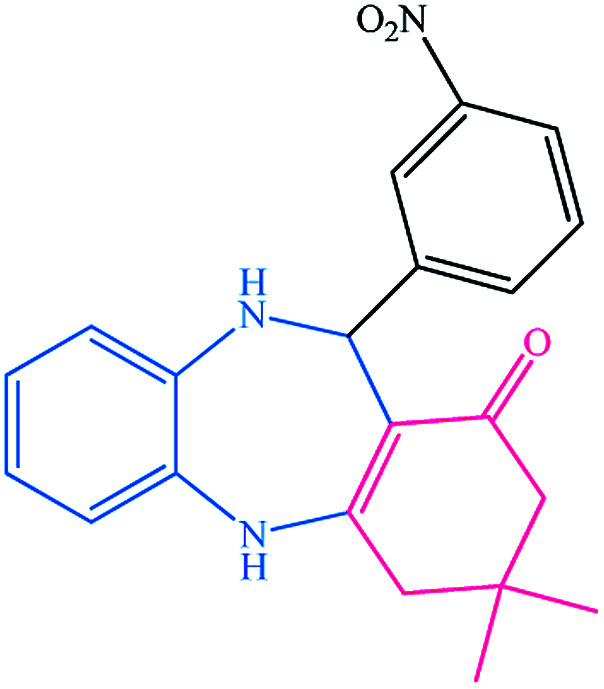	8	96	139–142 (ref. 38)
5	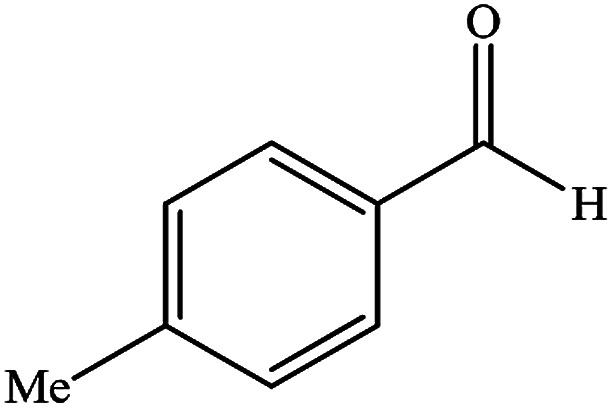	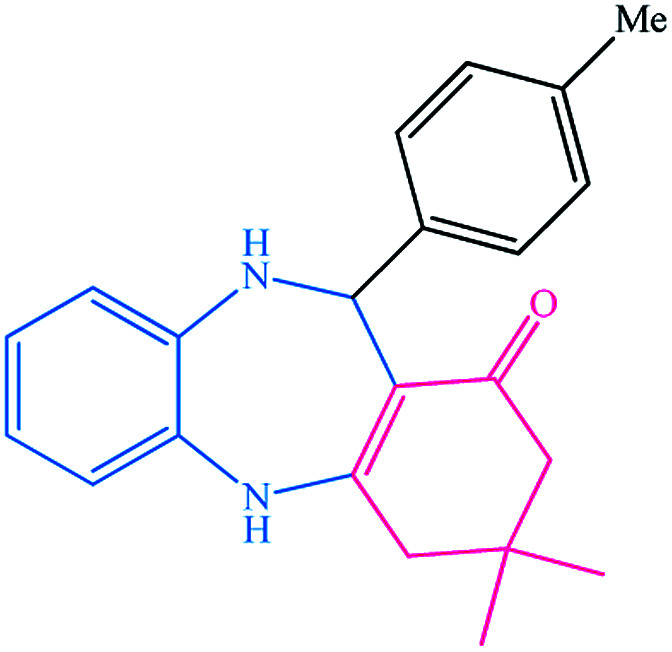	14	91	205–206 (ref. 37)
6	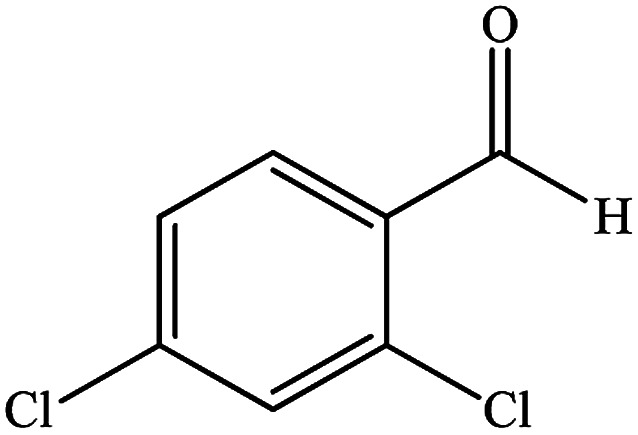	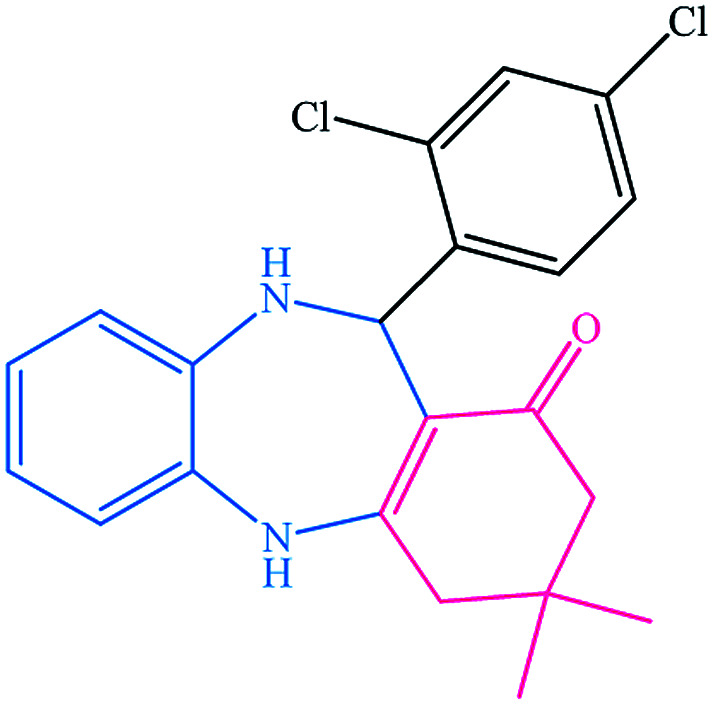	12	93	226–230 (ref. 32)
7	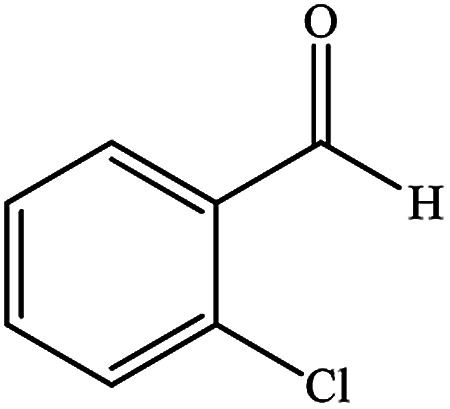	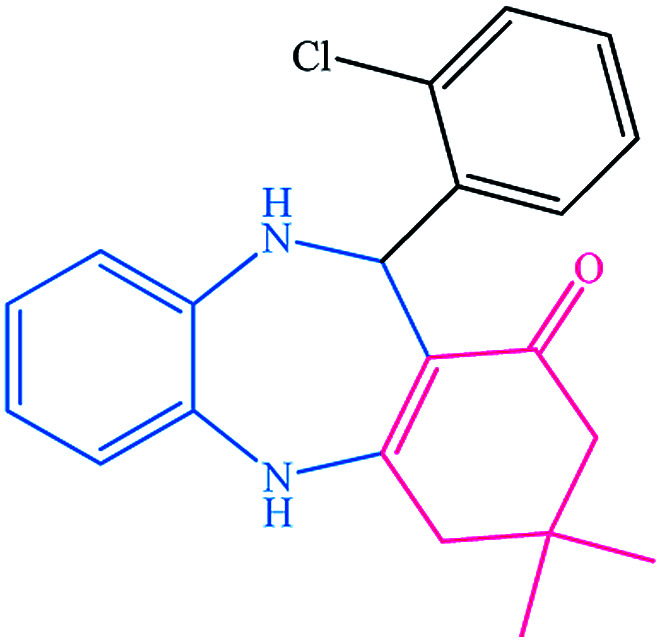	15	91	237–240 (ref. 39)
8	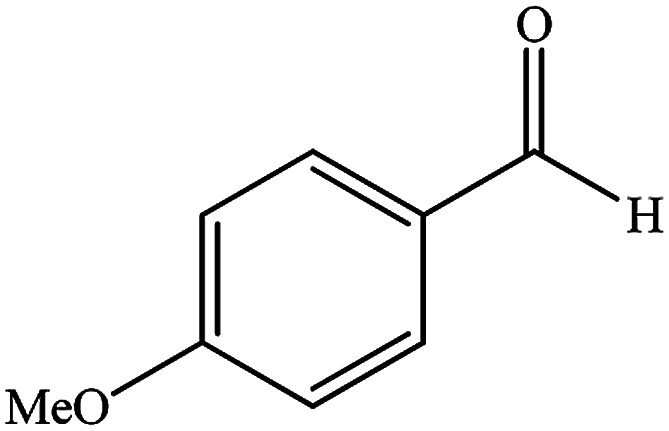	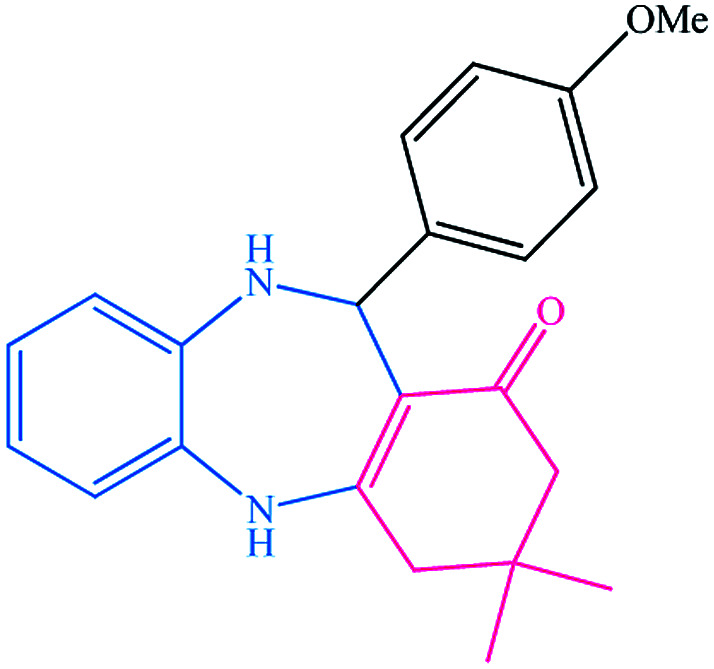	15	89	220–223 (ref. 40)
9	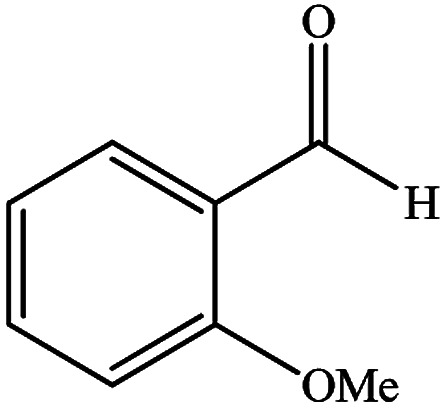	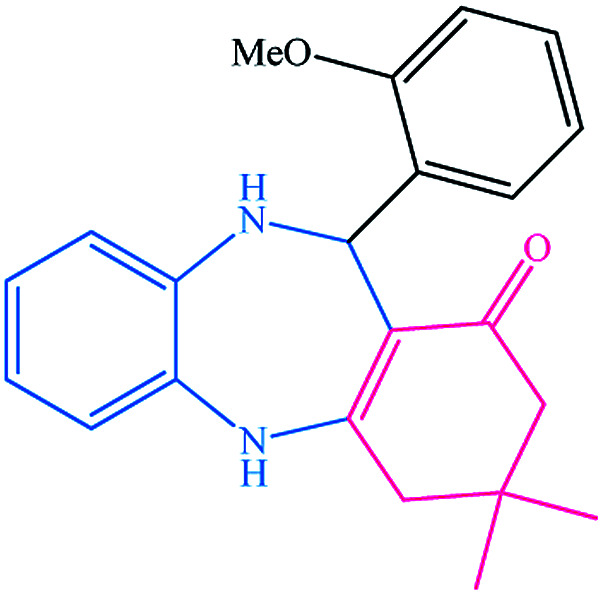	13	90	214–217 (ref. 32)
10	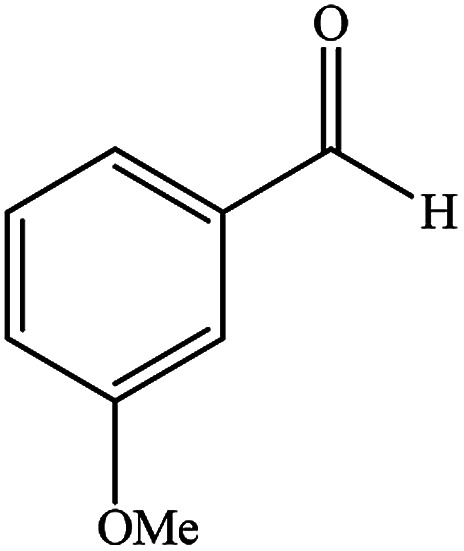	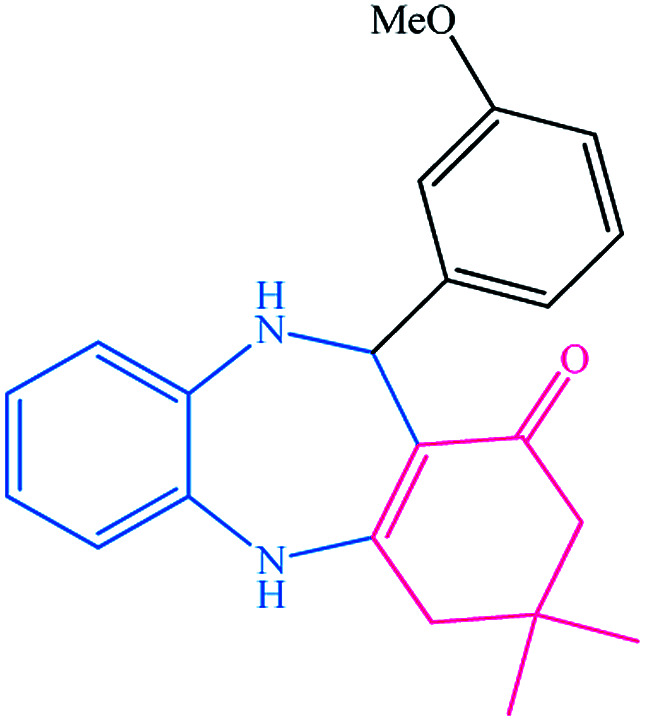	18	87	218–219 (ref. 32)
11	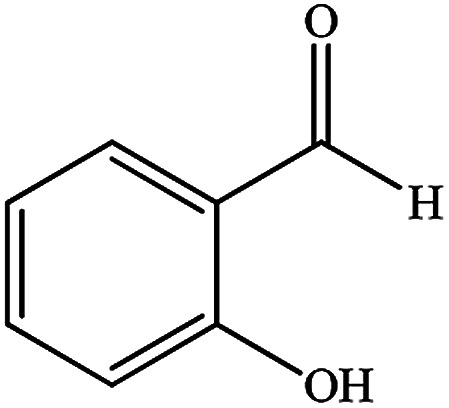	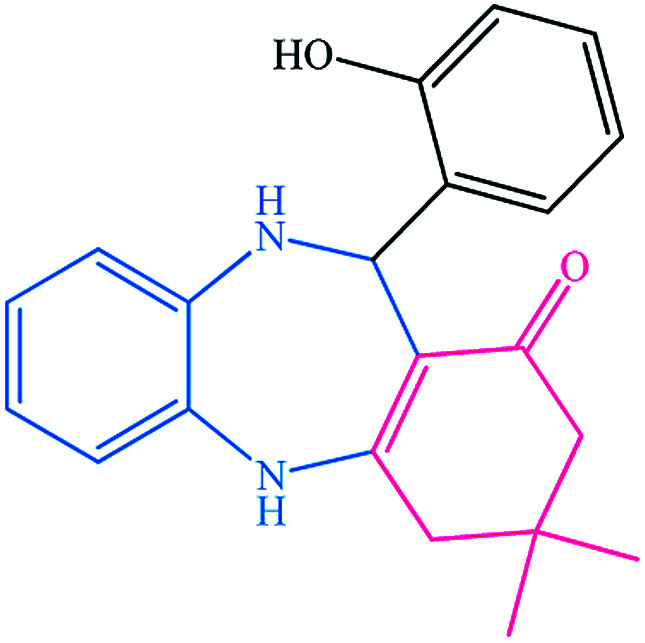	13	92	199–202 (ref. 32)
12	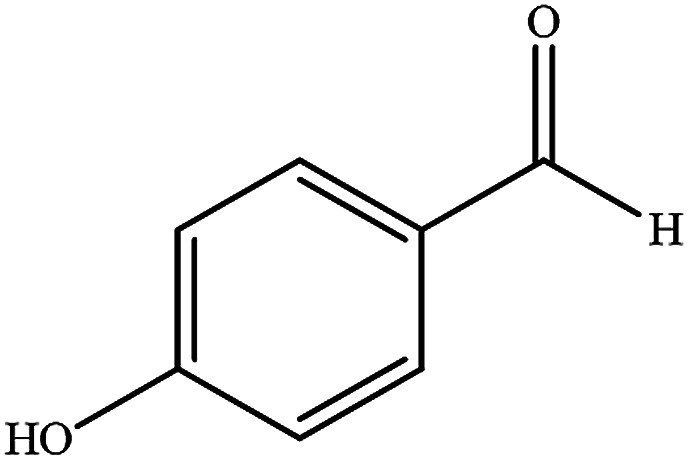	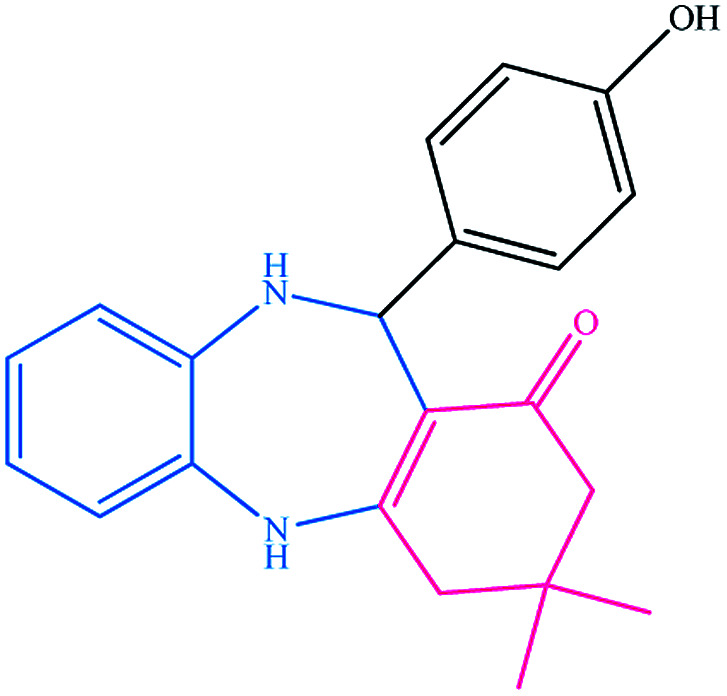	12	89	230–233 (ref. 32)
13	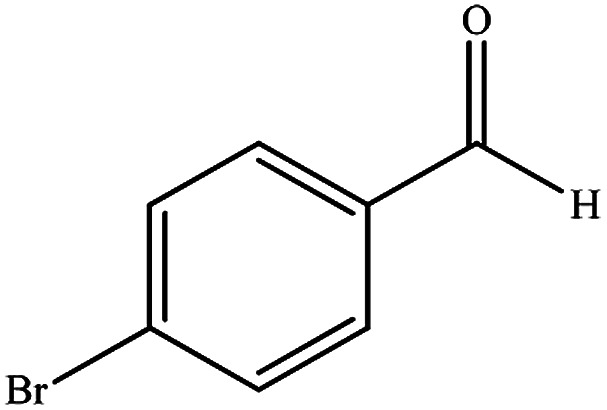	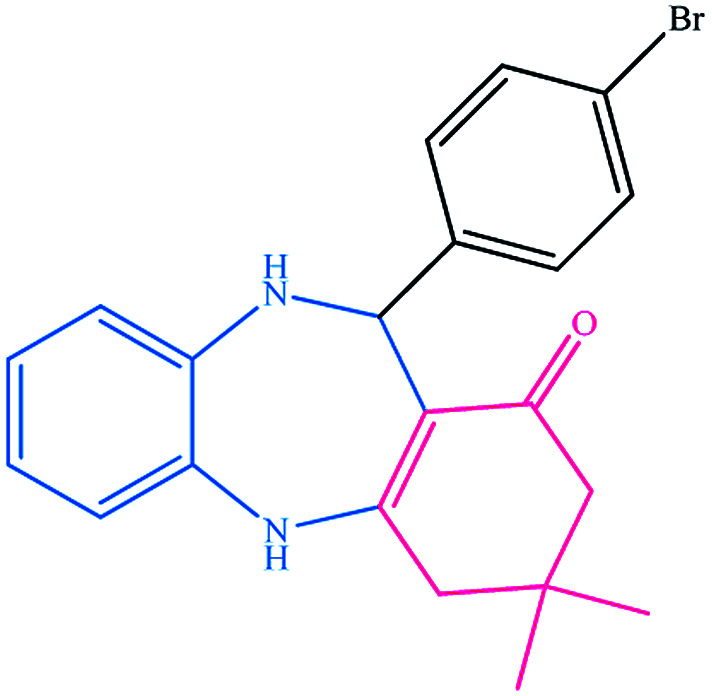	10	90	294–296 (ref. 40)
14	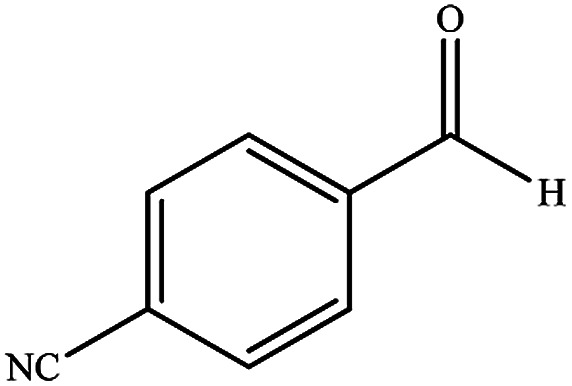	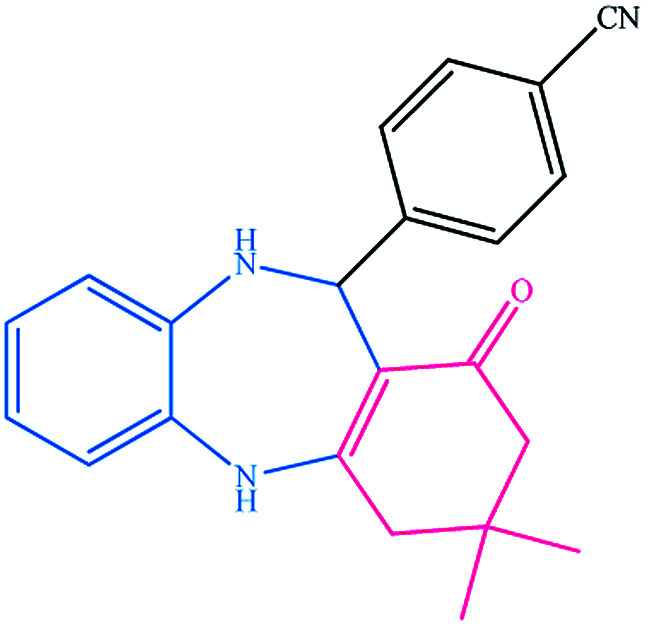	10	93	220–222 (ref. 40)

a All reactions were carried out in H_2_O (5 mL) under oil bath (60 °C) conditions.

**Scheme 3:**
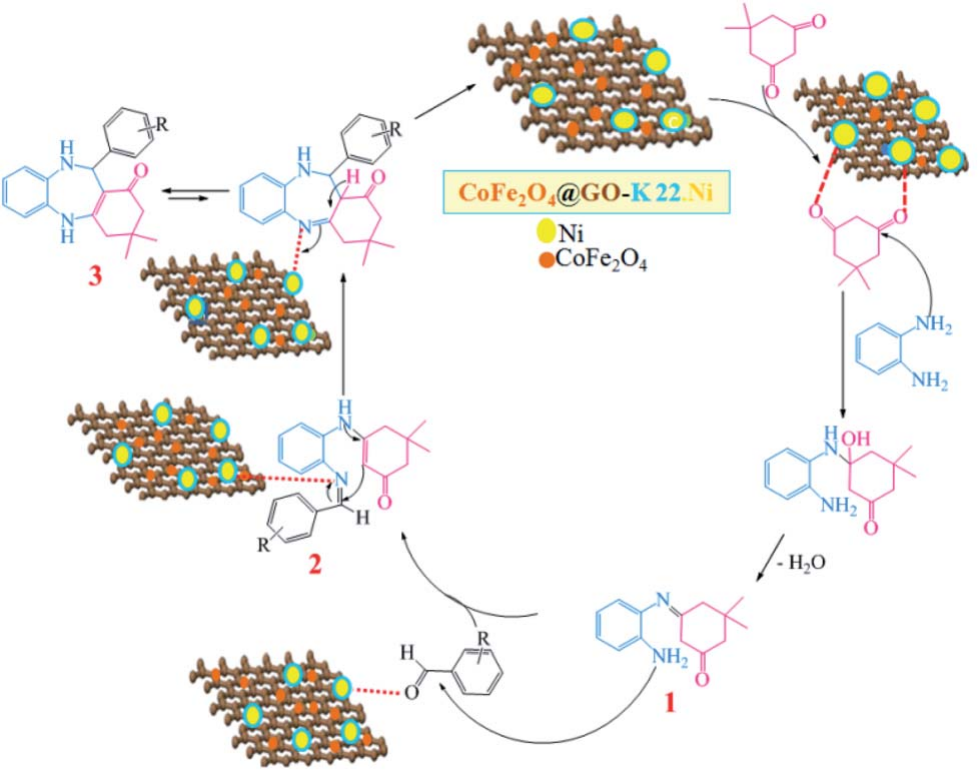
The plausible reaction mechanism for the synthesis of benzodiazepine derivatives.

After successful synthesis of benzodiazepine derivatives, a plausible reaction pathway has been suggested in Scheme 3.

Initially, the oxygen atoms of dimedone interact *via* lone pairs of electrons with nickel nanoparticles of the catalyst surfaces, and the NH_2_ groups of *o*-phenylenediamine attack the carbonyl group of dimedone with elimination of water molecule leading to imine intermediate 1. The amine group of intermediate 1 would then react with the activated carbonyl group of aldehyde to form the corresponding imine 2, which would undergo tautomerism, intramolecular cyclization, and proton transfer reactions to give product 3.

A comparison of this work with other reported methods is collected in Table 3. The results shows that this procedure is better to some previously reported methods in terms of easy of catalyst separation, yield, the amount of used catalyst and the reaction time.

**Table 3:** Comparing the catalytic activity of CoFe_2_O_4_@GO–K 22·Ni with the reported catalysts in multi-component reaction

Entry	Catalyst	Solvent	Time (min)	Yield (%)	Ref.
1	CoFe_2_O_4_@GO–K 22·Ni (0.03 g)/60 °C	H_2_O	10	95	This work
2	GO nanosheet (15 mg)/100 °C	H_2_O	30	95	40
3	NiO–SiO_2_ NCs (0.05 g), MW	Ethanol	10	98	32
4	Oxalic acid (40 mol%)/100 °C	H_2_O	120	94	41
5	TiO_2_ NPs (0.14 g)/20 °C	Ethanol	100	75	31

### 3.2. Recycling of CoFe_2_O_4_@GO–K 22·Ni

**Fig. 9:**
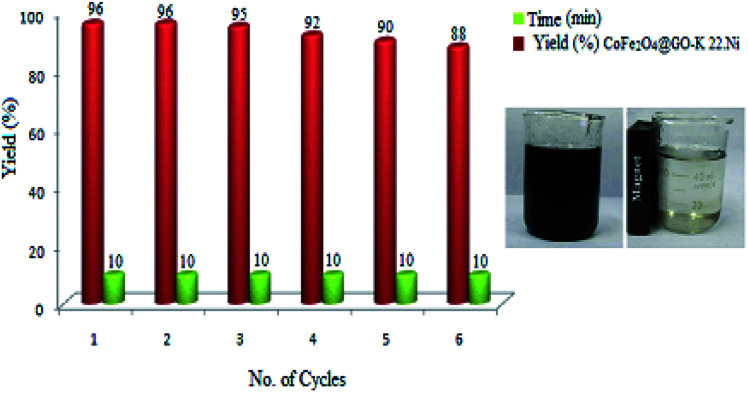
Recyclability of CoFe_2_O_4_@GO–K 22·Ni in the synthesis of 1,4-benzodiazepine.

For studying the recyclability of CoFe_2_O_4_@GO–K 22·Ni, the catalyst was isolated by an external magnet after the completion of each reaction run and washed several times with Et_2_O. The recovered catalyst was then reused in 6 cycles with minimal loss of activity (Fig. 9).

**Fig. 10:**
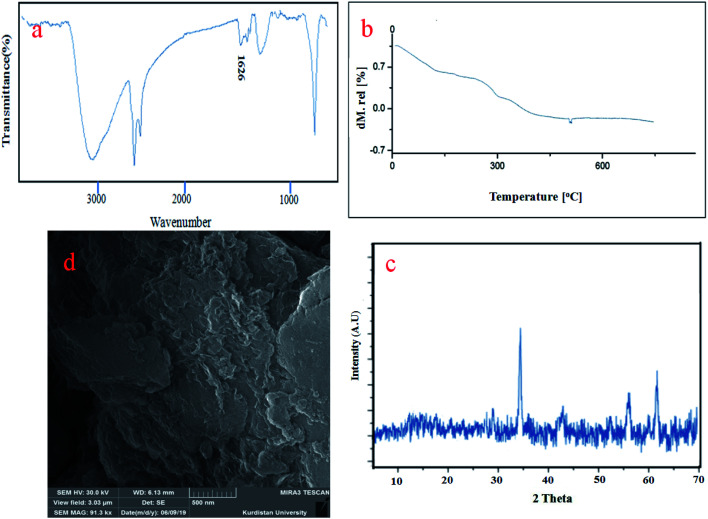
FT-IR (a), TGA (b), XRD (c), and SEM analyses of reused catalyst after six run.

To characterize the changes in the chemical structure of the catalysts following the sixth cycle, TGA, XRD, FT-IR and SEM analyses were carried out and the results are displayed below (Fig. 10). These analyses showed that CoFe_2_O_4_@GO–K 22·Ni nanocomposite maintained its chemical structure after the longevity tests.

The efficiency and activity of prepared catalyst was investigated by hot filtration test. The heterogeneity of the CoFe_2_O_4_@GO–K 22·Ni was examined by carrying out a hot filtration test using dimedone, *o*-phenylenediamine and benzaldehyde as model substrates. No nickel could be detected in the liquid phase using AAS and, more significantly, after hot filtration, the reaction of the residual mixture was completely stopped.

## 4. Conclusions

In this study we successfully reported an effective practice for the synthesis of efficient recoverable heterogeneous catalytic system, CoFe_2_O_4_@GO–K 22·Ni, achieved by anchoring Ni on the Kryptofix 22-modified magnetic nano-graphene oxide. The catalytic behavior of the catalyst was investigated as a recyclable system for the synthesis of 1,4-benzodiazepine. The introduced catalyst can promote the yields and reaction times over 6 repeated runs with very low leaching amounts of supported catalyst into the reaction mixture. The reaction conditions (H_2_O as solvent and without exclusion of air) coupled with the sustainability of the catalyst make the described heterogeneous catalyst highly desirable from the point of view of green chemistry. We expect that this new and practical protocol will be useful in pharmaceutical development and in academic research.

## Conflicts of interest

There are no conflicts to declare.

## Supplementary Material

RA-010-D0RA01671C-s001
