# Supplementary material for: A nickel nanoparticle engineered CoFe2O4@GO–Kryptofix 22 composite: a green and retrievable catalytic system for the synthesis of 1,4-benzodiazepines in water
Source: RSC Adv. 2020 Apr 16;10(26):15052–64. doi: 10.1039/d0ra01671c (PMC9052210; doi:10.1039/d0ra01671c)
Supplement: RA-010-D0RA01671C-s001 [file RA-010-D0RA01671C-s001.pdf]

Electronic Supplementary Material (ESI) for RSC Advances.
This journal is © The Royal Society of Chemistry 2020

Supplementary Information

Nickel nanoparticles engineered CoFe2O4@GO-kryptofix 22 composite: A green and retrievable catalytic system for the synthesis of 1,4-benzodiazepines in water

11-(phenyl)-3,3-dimethyl-2,3,4,5,10,11-hexahydro-1Hdibenzo[b,e][1,4]diazepin-1-one (Table 2, entry 1).

^{1}NMR (DMSO 400 MHz) δ (ppm) = 1.00 (s, 3H, CH_3), 1.02 (s, 3H, CH_3), 2.02 (d, 1H, J = 16.0 Hz, CH_2), 2.10 (d, 1H, J = 15.6 Hz, CH_2), 2.58 (s, 2H, CH2–C=O), 5.68 (d, 1H, J=5.6 Hz, C–H), 6.17 (d, 1H, J=6.0 Hz, N–H), 6.51 (d, 1H, J = 7.2 Hz, Ar), 6.54–6.61 (m, 2H, Ar), 6.90 (d, 1H, J= 7.2 Hz, Ar), 6.97-7.12 (m, 5H, Ar), 8.76(s, 1H, N–H); ^{13}C NMR (100 MHz, DMSO-d6) δ (ppm) = δ 27.8, 29.0, 33.2, 40.5, 49.9, 56.3, 110.6, 119.9, 120.4,120.9, 123.1, 126.3, 127.7, 128.2, 131.4, 139, 145.2, 155.3, 192.6.

11-(4-Chlorophenyl)-3,3-dimethyl-2,3,4,5,10,11-hexahydro-1H dibenzo[b,e][1,4]diazepin-1-one (Table 2, entry 3).

^{1}H NMR (DMSO 400 MHz) δ (ppm) = 1.02 (s, 3H, CH3), 1.08 (s, 3H, CH_3), 2.08 (d, 1H, J = 16.0 Hz, CH_2), 2.19 (d, 1H, J = 16.0 Hz, CH_2), 2.58 (s, 2H, CH_2–C=O), 5.66 (d, 1H, J=5.6 Hz, C–H), 6.19 (d, 1H, J=5.6 Hz, N–H), 6.51 (d, 1H, J = 7.2 Hz, Ar), 6.57–6.64 (m, 2H, Ar), 6.92 (d, 1H, J = 8.0 Hz, Ar), 7.07 (d, 2H, J = 8.4 Hz, Ar), 7.17 (d, J = 8.4 Hz, 2H, Ar), 8.82(s, 1H, N–H); ^{13}C NMR (100 MHz, DMSO-d6) δ (ppm) = δ 28.0, 28.8, 32.3, 44.5, 49.8, 55.9, 110.1, 120.3, 120.7, 121.1, 123.3, 128.1, 129.5, 130.1, 131.6, 138.8, 144.2, 155.4, 192.3

11-(4-methoxyphenyl)-3,3-dimethyl-2,3,4,5,10,11-hexahydro-1H dibenzo[b,e][1,4]diazepin-1-one (Table 2, entry 8).

^{1}H NMR (DMSO 400 MHz) δ (ppm) = 1.03 (s, 3H, CH_3), 1.09 (s, 3H, CH_3), 2.10 (d, 1H, J =16.0 Hz, CH2), 2.20 (d, 1H, J = 16.0 Hz, CH_2), 2.61 (s, 2H, CH2–C=O), 3.36 (s, 3H, Me), 5.72 (d, 1H, J=5.8 Hz, C–H), 6.32 (d, 1H, J=5.9 Hz, N–H), 6.52 (d, 1H, J = 8 Hz, Ar), 6.61–6.65 (m, 2H, Ar), 6.95 (d, 1H, J = 8.0 Hz, Ar), 7.24 (d, 2H, J = 8.6 Hz, Ar), 7.61 (d, J = 8.2 Hz, 2H, Ar), 8.90(s, 1H, N–H).

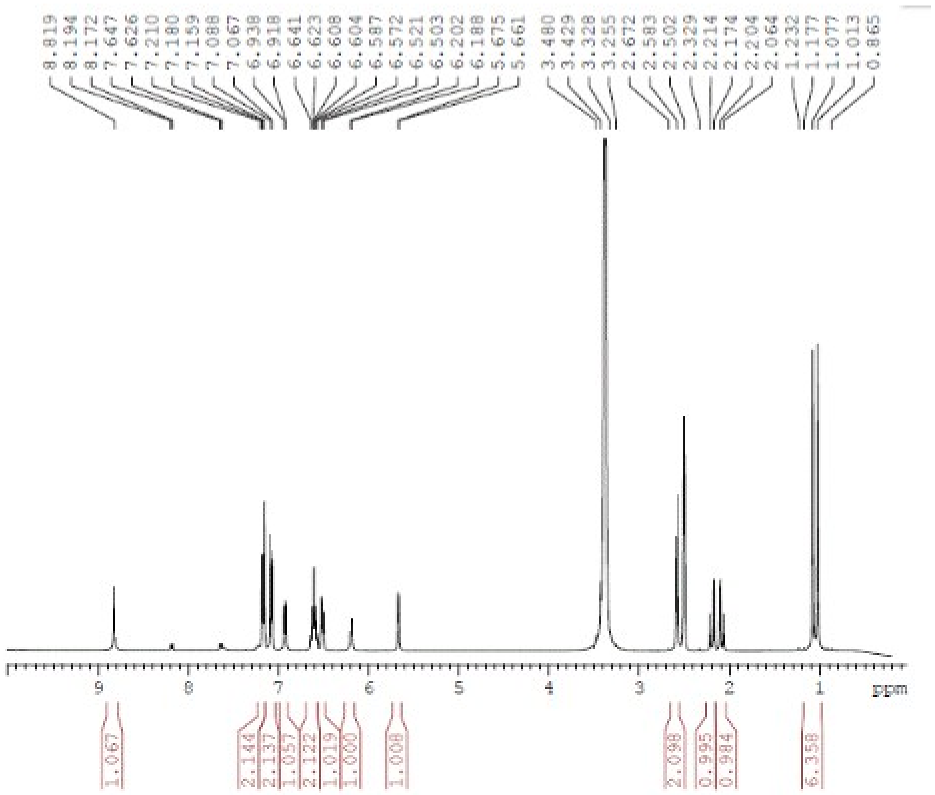

Fig 1 S. ^{1}HNMR: 11-(phenyl)-3,3-dimethyl-2,3,4,5,10,11-hexahydro-1H dibenzo[b,e][1,4]diazepin-1-one

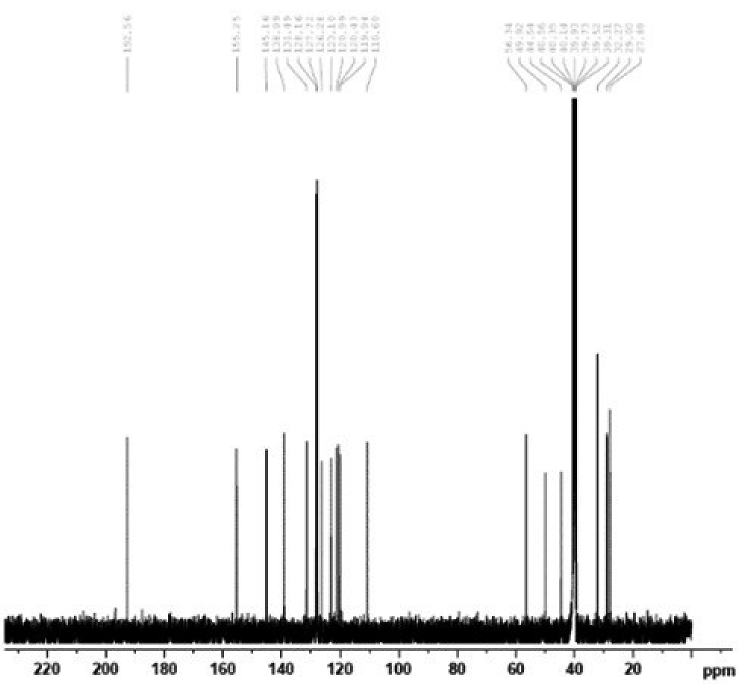

Fig 2 S.^{13}CNMR: 11-(phenyl)-3,3-dimethyl-2,3,4,5,10,11-hexahydro-1H dibenzo[b,e][1,4]diazepin-1-one

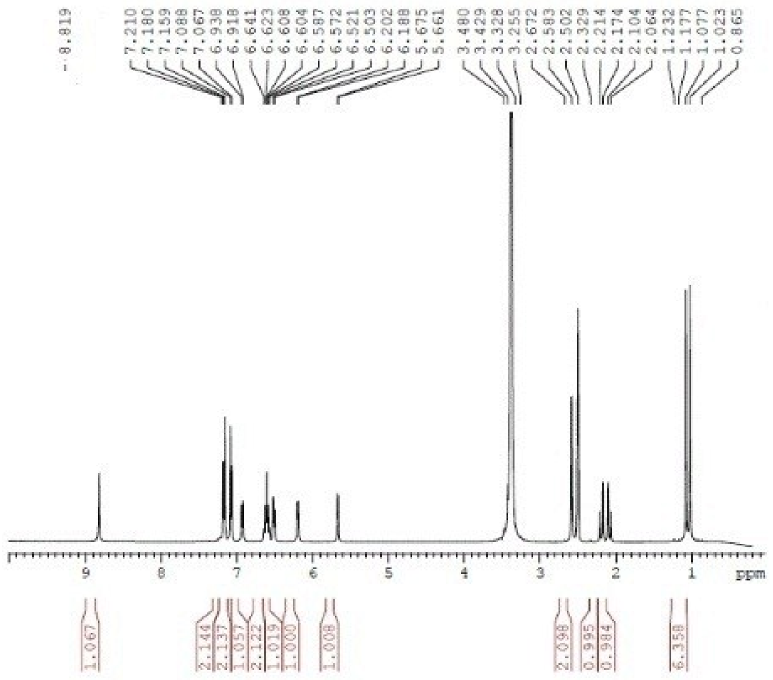

Fig 3 S.[1] HNMR:11-(4-Chlorophenyl)-3,3-dimethyl-2,3,4,5,10,11-hexahydro-1H dibenzo[b,e][1,4]diazepin-1-one

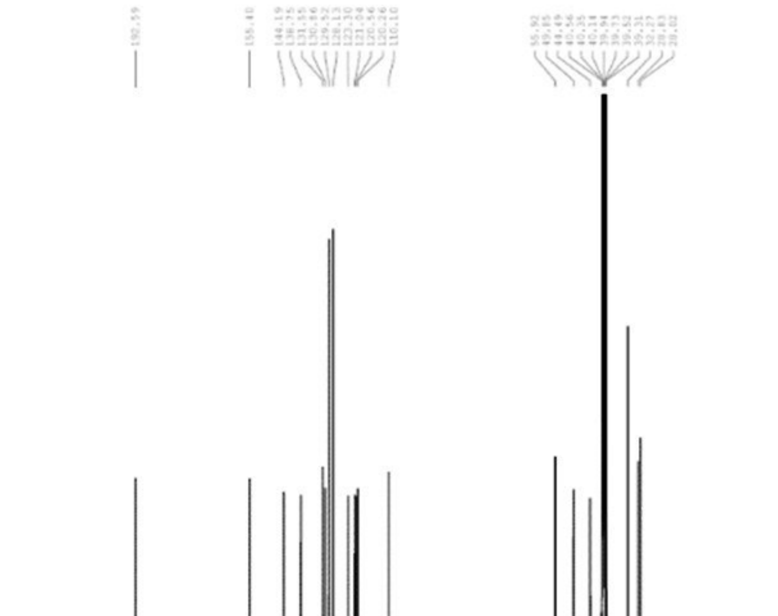

Fig 4 S. ^{13}CNMR:11-(4-Chlorophenyl)-3,3-dimethyl-2,3,4,5,10,11-hexahydro-1H dibenzo[b,e][1,4]diazepin-1-one

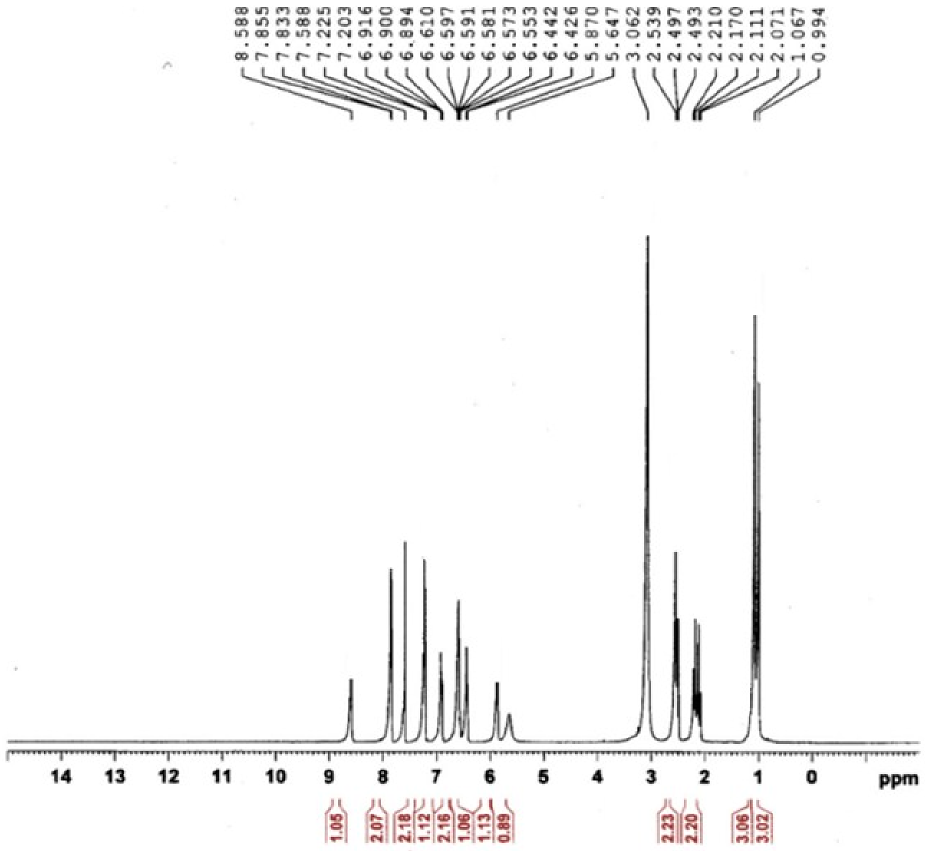

Fig 5 S. ^{1}HNMR:11-(4-Bromophenyl)-3,3-dimethyl-2,3,4,5,10,11-hexahydro-1H dibenzo[b,e][1,4]diazepin-1-one

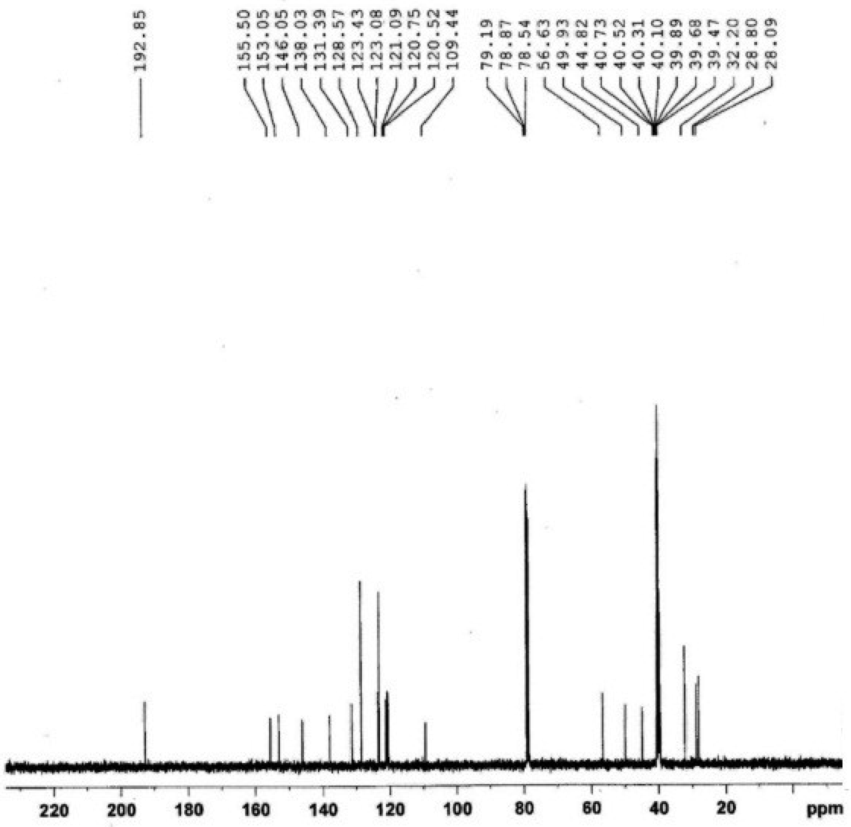

Fig 6 S. ^{13}CNMR:11-(4-Bromophenyl)-3,3-dimethyl-2,3,4,5,10,11-hexahydro-1H dibenzo[b,e][1,4]diazepin-1-one
